# Amylase Trypsin Inhibitors (ATIs) in a Selection of Ancient and Modern Wheat: Effect of Genotype and Growing Environment on Inhibitory Activities

**DOI:** 10.3390/plants11233268

**Published:** 2022-11-28

**Authors:** Emanuela Simonetti, Sara Bosi, Lorenzo Negri, Giovanni Dinelli

**Affiliations:** Department of Agricultural and Food Sciences, Alma Mater Studiorum—University of Bologna, 40127 Bologna, Italy

**Keywords:** wheat amylase-trypsin inhibitors (ATIs), ancient wheat, enzymatic assay, α-amylase inhibitory activity, trypsin inhibitory activity, modern wheat, environmental conditions, human nutrition

## Abstract

Wheat amylase-trypsin inhibitors (ATIs) are a family of plant defense proteins with an important role in human health for their involvement in allergies, celiac disease and non-celiac wheat sensitivity. Information about the differences in ATI activities among wheat genotypes and the influence of the growing environment is scarce. Therefore, ten selected wheat accessions with different ploidy level and year of release, previously characterized for their ATI gene sequences, were grown during three consecutive crop years at two growing areas and used for in vitro ATI activities. The contributions of the genotype and the crop year were significant for both activities. The hexaploid wheat genotypes showed the highest inhibitory activities. Einkorn had a peculiar behavior showing the lowest alpha-amylase inhibitory activity, but the highest trypsin inhibitory activity. It was not possible to observe any trend in ATI activities as a function of the release year of the wheat samples. The two inhibitory activities were differently affected by the growing conditions and were negatively correlated with the protein content. This information can be important in understanding the extent of variation of ATI inhibitory properties in relation to the wheat genotype and the growing environment and the impact of ATIs, if any, on human health and nutrition.

## 1. Introduction

Wheat amylase-trypsin inhibitors (ATIs) are a family of water-soluble proteins with low molecular weight and account for about 2–4% of total wheat protein [1]. Four groups of ATIs have been identified: three groups of alpha-amylase inhibitors are classified according to their degree of aggregation—monomeric (WMAI), homodimeric (WDAI) and heterotetrameric forms (WTAI)—and one group of trypsin inhibitors which are monomeric [2]. In modern hexaploid wheat, five sub-units of tetrameric inhibitors have been identified: CM1, CM2, CM3, CM16 and CM17. Tetrameric inhibitors are generally composed by one copy of either CM1 or CM2 sub-unit, plus one copy of either CM16 or CM17 sub-unit, plus two copies of CM3 sub-unit.

ATIs are accumulated at high levels in the endosperm of the grain kernel where they give an important contribution to plant defense against pests and pathogens [2]. They can inhibit amylases and/or trypsin or other proteases in many common parasites such as mealworms and mealy bugs from digesting starch and protein in wheat [3]. ATIs also function as storage proteins. With a more balanced amino acid composition compared to gluten proteins which are rich in glutamine and proline, ATIs make an important contribution to both seedling growth and human nutrition [4]. 

Regarding the impact on human health, ATIs are involved in bakers’ eczema, asthma and food allergy, [5] and, recently, they have gained an increasing interest for having a role in the etiology of celiac disease and non-celiac wheat sensitivity [6]. Studies carried out in vitro and in vivo in animals evidenced that ATIs can activate the toll-like receptor 4 (TLR4) complex playing a role in wheat as a main nutritional trigger of human and murine innate immunity [6,7,8]. ATIs can also act indirectly by suppressing the activity of digestive enzymes, namely trypsin and alpha-amylases, leading to a wide spectrum of gastrointestinal symptoms [9].

In recent years, there has been an increasing interest from consumers and farmers in landraces and old wheat varieties which are wheat species that remained unchanged over the last hundred years, in contrast to modern species released after 1950, i.e., after the phase commonly referred to as the “Green Revolution” [10]. Several studies highlighted their healthier nutritional profile and their higher antioxidant and anti-inflammatory properties compared to the modern wheat varieties. Although the mechanisms responsible for these beneficial effects are not completely understood, a different protein composition, a higher mineral content and a better phytochemical profile of ancient wheat genotypes seem to be the main determinants [11].

There are few and, in some cases, contrasting data in the literature about the differences in ATIs existing between different wheat genotypes and, in particular, between ancient and modern wheat varieties. Data concerning the inflammatory potential of ATIs (determined as pro-inflammatory cytokine release in TLR4-responsive mouse and human cell lines) from different wheat varieties showed that modern hexaploid wheat contained higher ATI inflammatory activity than some ancient variants like diploid (einkorn) and tetraploid wheat (emmer, khorasan wheat) or older hexaploid variants like spelt [8]. Regarding the evaluation of in vitro ATI inhibitory activities, recent studies did not find differences between ancient and modern wheat cultivars [9,12]. However, some of these studies were missing information about the growing conditions of the tested wheat varieties; moreover, the ATI gene sequence diversity among the wheat varieties tested was not known.

In the present study, ten selected wheat accessions with different ploidy level and year of release, previously characterized for their ATI gene sequences [13], were grown during three consecutive crop years at two growing areas (Italy and USA) and used for in vitro alpha-amylase and trypsin inhibitory activities with the aim to find possible differences among the wheat genotypes. Correlations between inhibitory activities, growing environment and genetic background of the wheat genotypes have been investigated and discussed. To the best of our knowledge, this is the first study investigating ATI activities of ancient and modern wheat genotypes with different ploidy levels, previously characterized for their ATI gene sequences and grown in different environments and in different crop years.

## 2. Results and Discussion

### 2.1. Characterization of Grain Samples

Ten selected samples belonging to different *Triticum* species were grown under organic farming management for three consecutive crop years at two locations in the USA and in Italy. At the American location, it was not possible to harvest Peliss wheat in 2016 and 2017 and emmer harvested in 2016 was not included in the analyses because it was destroyed while in storage.

**Figure 1:**
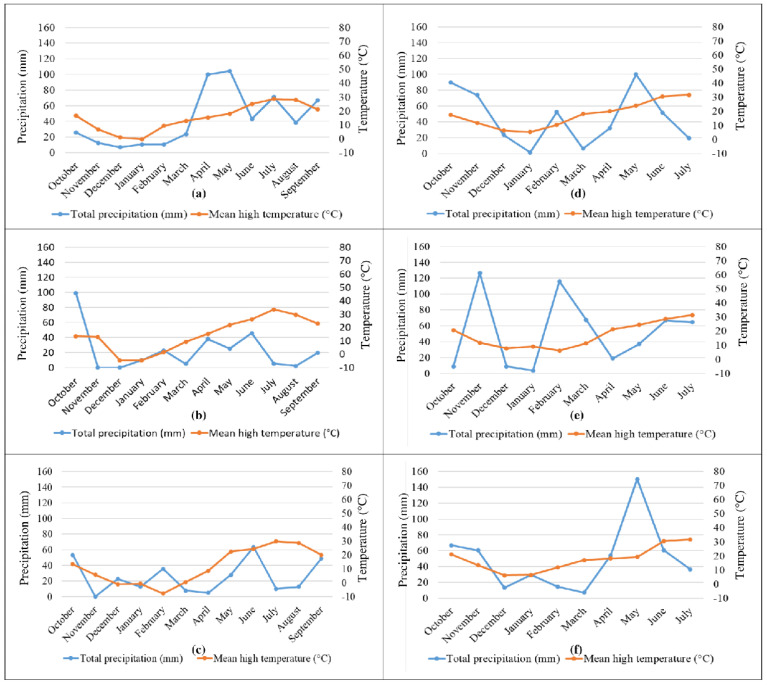
Total precipitation and mean temperatures over the cultivation years for 2015–2016 (**a**), 2016–2017 (**b**) and 2017–2018 (**c**) in the USA and for 2016–2017 (**d**), 2017–2018 (**e**) and 2018–2019 (**f**) in Italy.

The meteorological data (temperature and rain) recorded at both sites are shown in Figure 1.

All the wheat genotypes were tested for their protein contents and levels of mycotoxins (DON), and results are shown in Table 1.

In general, samples grown in the USA showed higher protein contents than those cultivated in Italy. This agrees with the fact that, compared to Italy, in Montana (USA) the average wheat yields are typically lower and the protein contents are higher [14]. All the levels of mycotoxins (DON) measured in this study were below the maximum limit allowed both for raw material durum wheat and oat (1750 ppb) and for the other raw material cereals (1250 ppb) (EU legislation n. 1881/2006). However, in the USA during the first crop year (2015–2016), there were values of DON content higher than the detection limit of analysis (200 ppb) in some samples, likely as consequence of more rainfall in the spring and summer registered in 2016 compared to the same period in 2017 and 2018. Similarly, in Italy the precipitation in spring was most abundant in 2019, and this resulted in positive DON levels in 8 out of 10 wheat genotypes harvested in 2019, all of which were below the EU legislation limit.

### 2.2. Alpha-Amylase Inhibitory Activity Assay

The results of the alpha-amylase inhibitory activity showed that there was high variability among the ten wheat genotypes analyzed (Table 2).

The general linear model (GLM) highlighted that the contribution of the fixed (genotype) and the random (year) factors, as well as their interactions, to the variability of the alpha-amylase inhibitory activity was significant in both growing areas. Similarly, another study showed that inhibitory activities against human saliva alpha-amylase in two cultivars of *T. aestivum*, triticale and rye were significantly affected by the variety, genus and year of harvest as sources of changes [15]. However, they could not highlight an effect of the year of harvest in relation to the wheat variety to the variability of the inhibitory activities.

In this study, the mean values for the alpha-amylase inhibitory activity ranged between 1.12% and 66.63%, with the lowest activity observed in einkorn samples in both growing areas. In general, except for emmer, the hexaploid wheat genotypes showed higher inhibitory activities than tetraploid and diploid wheat genotypes. Interestingly, these results are supported by the ATI gene sequences obtained previously from the same samples used in this study [13]. In fact, considering the WDAI (wheat homodimeric amylase inhibitors) gene sequences, the haplotypes showing a perfect homology with the three inhibitor spots important for the human alpha-amylase inhibitory activity [16] were present only in hexaploid wheat genotypes which also showed the highest alpha-amylase inhibitory activity in this study. The important contribution of WDAI to the total inhibitory activity is known in the literature. A previous study showed, in fact, that WDAI 0.19, even if consisted of 24.9% of total wheat albumin, contributed approximately 80% to the inhibiting activities against human alpha-amylase [17].

Considering the results of the alpha-amylase inhibitory activities, it was not possible to highlight differences between ancient and modern wheat genotypes. The same conclusions were reported by Gelinas et al. [9] who also highlighted that most of the hexaploid wheat cultivars had higher alpha-amylase inhibitory activity than tetraploids. Interestingly, within the common wheat genotypes, most of the hard red spring wheat tested by Gelinas et al. had higher alpha-amylase inhibitory activity than the hard red winter wheat. However, they did not have any information about the growing conditions and the agronomic performance of the tested wheat genotypes. In this study, the hard red winter common wheat genotypes (Turkey Red and Judee) showed higher activities than the hard red spring common wheat genotypes (Marquis and Vida) in the USA where a winter and a spring cycle, respectively, were used. Differently, in Italy where a winter cycle was used for all the wheat genotypes, Marquis showed a similar inhibitory activity than Turkey Red and Judee, but Vida seemed not to be influenced by the different growing cycle adopted in Italy and showed similar inhibitory activities as in the USA.

As mentioned above, the lowest activity was observed in einkorn samples in both growing areas with values much lower than the other wheat samples or even close to zero. These results agree with previous studies based on immunological, mass spectrometry and colorimetric tests [18,19,20,21]. It was previously suggested that the apparent absence of alpha-amylase inhibitory activity of einkorn could be explained by the fact that the corresponding coding genes might be expressed at very low level or even silenced, perhaps as a result of gene mutations that prevent the translation into the mature protein [22]. This hypothesis is confirmed by the gene sequencing results obtained previously from the same einkorn sample used in this study [13]. In fact, Simonetti and colleagues could not find any WTAI-CM3 gene sequence in einkorn, and, consequently, it should not be able to produce tetrameric alpha-amylase inhibitor proteins. Moreover, all the WDAI gene sequences of einkorn showed an insertion of C at position 160 which resulted in a premature stop codon which caused the impossibility to synthesize the correct WDAI mature protein. Lastly, einkorn showed functional WMAI gene sequences, but it is known that WMAI proteins weakly inhibit the human alpha-amylases [23]. On the other hand, WMAI proteins are highly active against alpha-amylase of Tenebrio molitor, and this could be related to the well-known high resistance of einkorn to pest and disease [21]. So, the results obtained in this study regarding the alpha-amylase inhibitory activity of einkorn are supported by the corresponding ATI gene sequences.

**Figure 2:**
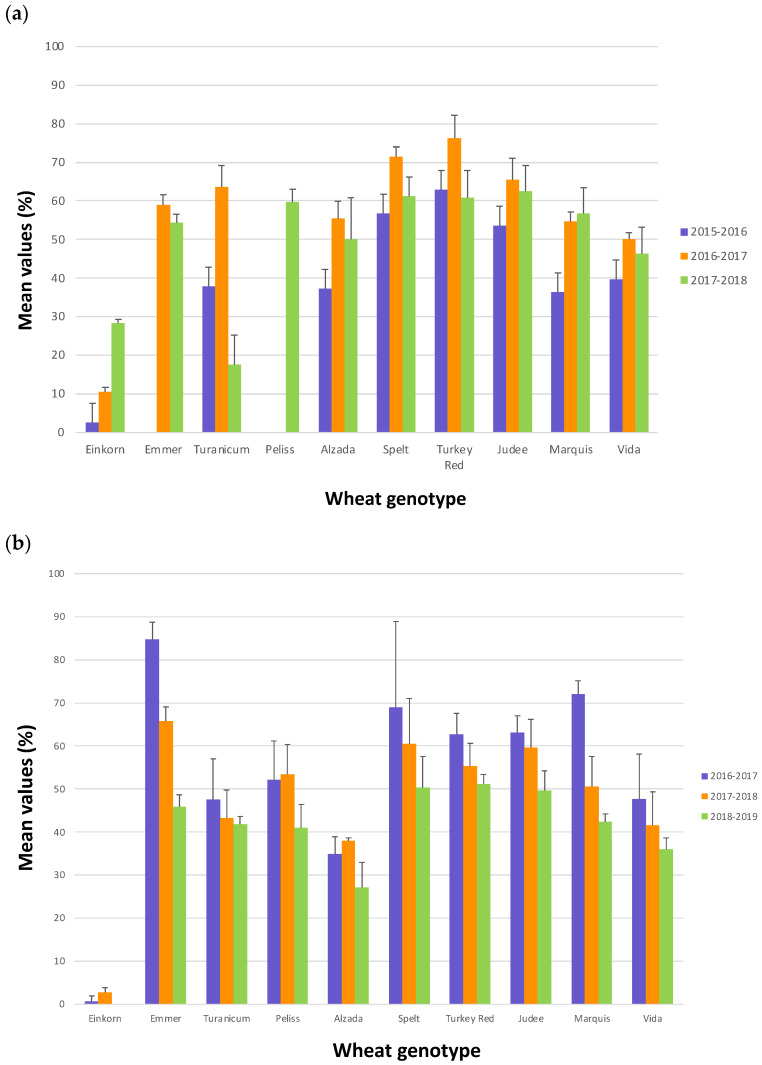
Mean values of the alpha-amylase inhibitory activity for each genotype in the three crop years. Samples grown in the USA (**a**); samples grown in Italy (**b**). The bars in the graph indicate the standard error (SE).

The mean values for each genotype in the three crop years are shown in Figure 2a (USA) and Figure 2b (Italy).

In the USA, 2016–2017 and 2017–2018 crop years showed higher alpha-amylase inhibitory activities than 2015–2016. All the wheat genotypes showed a similar trend among the three crop years with the exceptions of einkorn and turanicum samples which significantly increased and decreased, respectively, their activities in 2017–2018. As it is shown in Figure 1, in the USA there was more rainfall in spring and summer in 2016 compared to the same period in 2017 and 2018. More specifically, in May and July 2016 there was a double amount of precipitation compared to the averages reported in that area [24]. Considering that the last months before harvest are crucial for the kernels’ filling, it is interesting to note that the higher rainfall during the period May–August in 2016, compared to the same period in 2017 and 2018, corresponded to the lowest level of alpha-amylase inhibitory activity.

In Italy, the highest alpha inhibitory activities were in 2016–2017 and the lowest in 2018–2019. Overall, there were not samples showing a different trend compared to the average. The precipitation was most abundant in the last months before the harvest in 2019 (period April–July). In particular, the amount of precipitation in May was double compared to the averages for that area [25], and as seen in the USA, in this year the wheat samples showed the lowest levels of alpha-amylase inhibitory activity.

In both growing areas, higher precipitation resulted in higher DON content in wheat genotypes, but corresponded to lower alpha-amylase inhibitory activities, differently from what could be expected for a class of protein involved in plant defense. However, it should be taken into consideration that it is known in the literature that each type of ATIs shows different specificities against different enzymes [26], and in this study the inhibitory properties have been studied against mammalian enzymes.

Regarding the mean high temperatures for the same period analyzed for precipitation, the lowest values were registered in 2016 in the USA and in 2019 in Italy (Figure 1) which corresponded to the lowest levels of alpha-amylase inhibitory activity, highlighting a positive trend with these activities in both growing areas.

**Figure 3:**
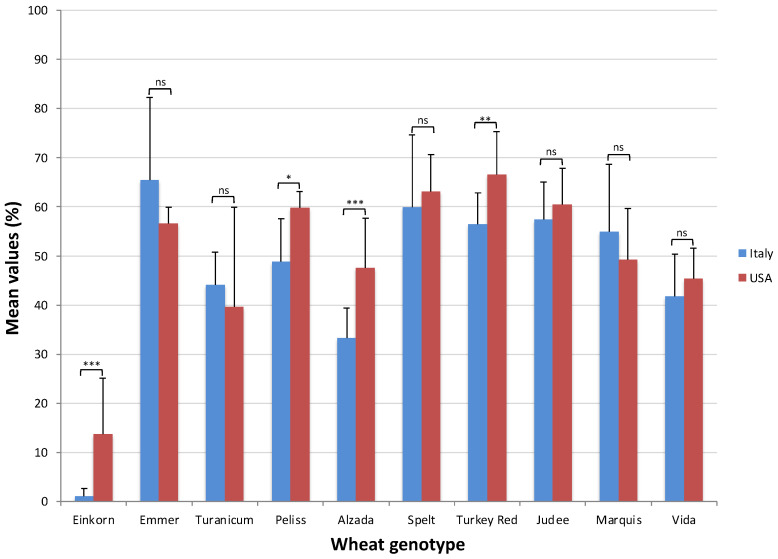
Differences between the mean values of alpha-amylase inhibitory activity in the USA and in Italy for each wheat genotype. Significance level: * *p* < 0.05; ** *p* < 0.01; *** *p* < 0.001. ns, not significant.

The two cultivation areas affected the wheat genotypes differently. Einkorn, Peliss, Alzada and Turkey Red all showed significantly higher alpha-amylase inhibitory activities in USA (Figure 3).

There were negative correlations between the protein content and the alpha-amylase inhibitory activities both in the USA (r = −0.626; *p* < 0.01) and in Italy (r = −0.526; *p* < 0.01). This agrees with Prandi et al. [27] who previously observed that locations that yielded more protein content consistently produced lower amounts of CM3 ATI.

### 2.3. Trypsin Inhibitory Activity Assay

As for the alpha-amylase inhibitory activities, the trypsin inhibitory activities showed a high variability among the ten wheat genotypes analyzed (Table 3).

The general linear model (GLM) showed that the contribution of the fixed (genotype) and the random (year) factors to the variability of the trypsin inhibitory activity were significant, but not their interactions. Previously Piasecka et al. [15] showed that inhibitory activities against bovine trypsin in two cultivars of T. aestivum, triticale and rye were statistically significant in all sources of changes (variety, genus, year of harvest as well as interaction between them).

The mean values of the trypsin inhibitory activity ranged between 18.61% and 100%, with the lowest activity observed in the Peliss sample and the highest activity observed in the einkorn sample in both growing areas and in all three crop years.

After einkorn, the hexaploid wheat genotypes Turkey Red, Judee and Marquis showed the highest inhibitory activities in both growing areas. Moreover, as for the alpha-amylase inhibitory activity, it was not possible to observe any trend as a function of the release year of wheat samples. These results are in line with the ATI gene sequence analysis results on the same wheat samples [13] and with previous studies which evaluated the ATI concentration in ancient and modern wheat. D’Amico et al. [28] used a new RP-HPLC method to determine the quantitative levels of ATIs in Austrian wheat varieties from the 19th century up to the present day and found a high variation among the samples. However, they could not highlight any correlation between ATI content and the year of release of the samples. Another study [29] determined the content of eight ATI proteins and the total ATI of 149 European old and modern bread-wheat cultivars grown at three different field locations, and also in this case it was not possible to find any trends regarding ATI content in wheat cultivars and their breeding history. All these cited studies suggest that the beneficial effects of ancient wheat on human health evidenced in recent studies are likely linked to other characteristics of these grains other than their ATI activity.

The hard red winter common wheat genotypes (Turkey Red and Judee) showed higher activities than the hard red spring common wheat genotypes (Marquis and Vida) in the USA where they were cultivated on a winter and on a spring cycle, respectively. However, in Italy where all the genotypes were cultivated on a winter cycle, Marquis showed a similar inhibitory activity as Turkey Red and Judee, but Vida seemed not to be influenced by the different growing cycle adopted in Italy and showed similar inhibitory activities as in the USA. Interestingly, the same behavior of Marquis and Vida samples were observed in this study for the alpha-amylase inhibitory activity. So, besides the genotype, it seems that also the crop cycle can influence the expression of these inhibitors. However, further studies on more samples are needed to confirm this hypothesis.

Data regarding trypsin inhibitory activity of wheat are very scarce in the literature. A few studies compared trypsin inhibitory activity in wheat with other cereals and legumes and showed that it is much lower in wheat than in rye, triticale and soybean [15,30]. Regarding more specifically the genus Triticum, Priya et al. [26] found large variation in trypsin inhibitory activity against insect and bovine trypsin in 54 genotypes of *T. aestivum.*

The absence of correlation between the release year of wheat samples and the trypsin inhibitor activity and the high activities observed in einkorn samples are in line with what is observed in a recent paper where the authors analyzed the ATI concentrations and trypsin inhibitor activities in a set of different Triticum species [12]. They also obtained the lowest activity with emmer samples unlike this study where emmer sample showed a low activity only when grown in the USA with a spring cycle. They could not find any correlation between the ATI concentrations and the trypsin inhibitor activities in einkorn and they hypothesized the presence of other proteins showing high affinity to trypsin. In fact, previously, Taddei et al. [31] and then Sielaff et al. [32] found specific einkorn trypsin inhibitors (ETIs), not detected in durum and soft wheat, that can explain the high trypsin inhibitory activity in einkorn despite the low levels of detected ATIs. The ATI gene sequences determined by Simonetti et al. [13] highlighted a separate genetic profile of einkorn from the other nine wheat samples, and this can support the distinct inhibitory activities of einkorn (lowest alpha-amylase and highest trypsin inhibitory activities) obtained in this study using the same wheat sample.

**Figure 4:**
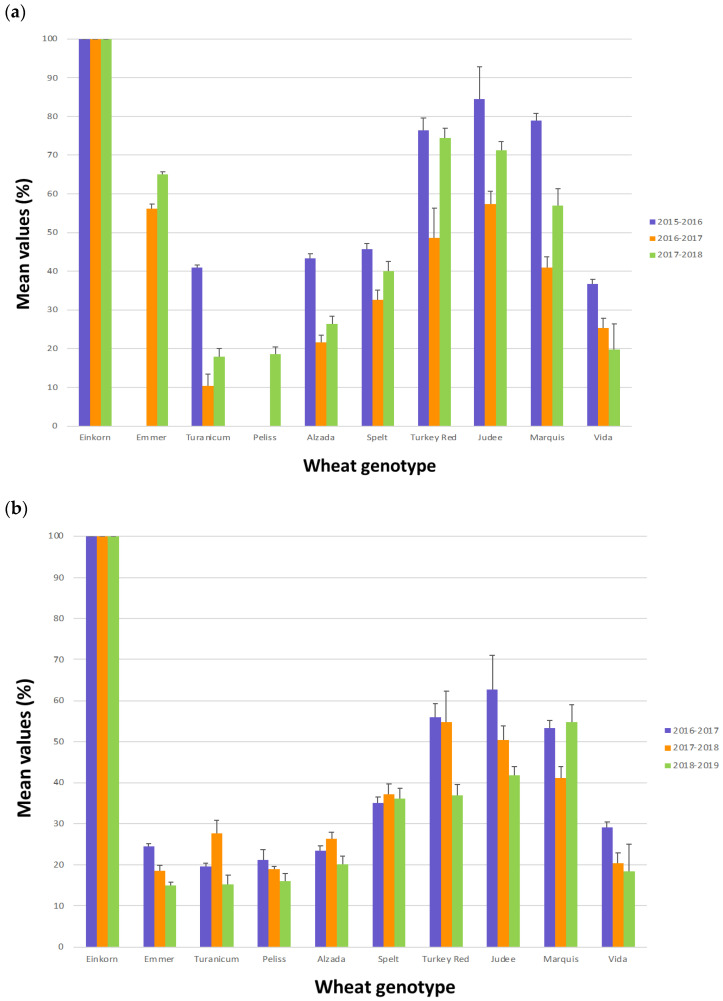
Mean values of the trypsin inhibitory activity for each genotype in the three crop years. Samples grown in the USA (**a**); samples grown in Italy (**b**). The bars in the graph indicate the standard error (SE).

Regarding the interaction of the trypsin inhibitory activity with the crop year, as shown in Table 3, in the USA the 2015–2016 crop year showed the highest and 2016–2017 the lowest trypsin inhibitory activities. In Italy, the highest trypsin inhibitory activities were in 2016–2017 and the lowest in 2018–2019. All the genotypes showed a similar trend among the three crop years both in the USA and in Italy (Figure 4a,b).

Considering the same period analyzed for alpha-amylase inhibitory activities, the highest precipitation corresponded to the lowest trypsin inhibitory activity in Italy (2018–2019), but to the highest trypsin inhibitory activity in the USA (2015–2016). Similarly, the lowest values for the mean high temperatures corresponded to the lowest trypsin inhibitory activity in Italy (2018–2019), but to the highest trypsin inhibitory activity in the USA (2015–2016) (Figure 1 and Table 3). So, the trypsin inhibitory activities in Italy showed the same trends with respect to rainfall and high mean temperature as seen for the alpha-amylase inhibitory activities in both areas, but in the USA these trends were opposite.

**Figure 5:**
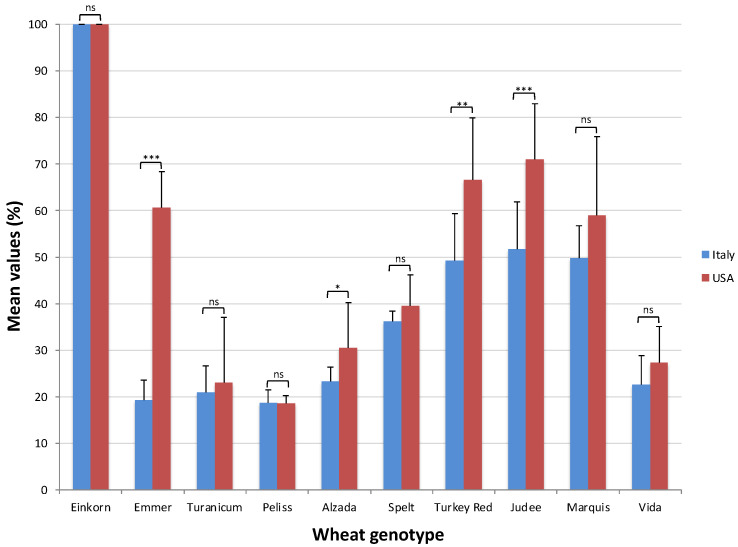
Differences between the mean values of trypsin inhibitory activity in the USA and in Italy for each wheat genotype. Significance level: * *p* < 0.05; ** *p* < 0.01; *** *p* < 0.001. ns, not significant.

Genotypes responded differently to the growing areas; in particular emmer, Judee, Turkey Red and Alzada showed significantly higher trypsin activities in the USA, even three times more considering emmer (Figure 5).

As for the alpha-amylase inhibitory activities, there were negative correlations between the protein content and the trypsin inhibitory activities both in the USA (r = −0.273; *p* < 0.01) and in Italy (r = −0.489; *p* < 0.01).

### 2.4. ATI Inhibitory Activity, Genotype and Growing Environment

To highlight the contribution of genotype and crop year and their interaction to the variability of alpha-amylase and trypsin inhibitory activity, the mean square values of each factor (obtained from general linear model elaborations) have been reported in Table 4 and expressed as the percentage values of the total mean square (excluding mean square of error factor).

The wheat genotype had the most effect on the inhibitory activities of both locations, representing 55–65% of the variability, which was higher for trypsin inhibitory activity especially in Italy where it exerted a predominant effect (91.5%). Genotype × Year interaction was small but significant for alpha-amylase inhibitor activity and non-significant for trypsin inhibitory activity. This indicates a higher stability of trypsin inhibitors which seem to be less influenced by the climatic conditions compared to alpha-amylase inhibitors, especially in Italy. A possible explanation could be provided by the type of soil in the two test areas. In fact, the soil in Italy was clay–loam which is known to be able to retain moisture and so to keep more constant the moisture level in the soil unlike the soil in the USA which was sandy–loam and so prone to moisture loss. A more constant level of moisture in Italy could have stimulated less enzyme activity, especially of trypsin inhibitors which were less affected by the environment than alpha-amylase inhibitors in both Italy and the USA. As a result, the genotype had a significantly higher contribution on the variability of trypsin inhibitory activity in Italy.

Besides genetic factors, the results shown in Table 4 suggest that also the crop year gave an important contribution to the variability of ATI activity in the wheat grains. In this study, different climatic conditions corresponded to different inhibitory activities of wheat genotypes. In both cultivation areas, higher precipitation and lower mean-high temperatures were observed during the spring and summer of the year characterized by the lowest alpha-amylase inhibitory activities, while there were opposite trends for the two growing areas regarding trypsin inhibitory activity.

Figure 3 and Figure 5 showed that the mean values of both alpha-amylase and trypsin inhibitory activity were lower in Italy compared to the USA for most wheat genotypes. As explained above, the soil in Italy was clay–loam which is known to be able to retain moisture unlike the soil in the USA which was sandy–loam. Moreover, Figure 1 shows in the months just before harvest the curve related to mean high temperatures is always over the curve of precipitation in the USA unlike in Italy. So, considering both the soil types and the meteorological data, it is likely that in Italy there was higher availability of water compared to the USA for the three years of the study, and that corresponded to lower ATI activities in Italy, confirming again the negative trend between ATI activity and precipitation in Italy.

A previous study also highlighted a negative correlation between the monthly precipitation (particularly in January and in March) and the alpha-inhibitory activities in two cultivars of T. aestivum against the hog pancreas alpha-amylase, but not against human alpha-amylase and insects α-amylase [15]. Other studies showed that high-temperature and water-deficit stress conditions can influence the ATI levels in seed, indicating a possible stress-related function of ATIs from seed germination through seed development [33,34,35]. Moreover, another study showed that wheat endosperm expressed alpha-amylase inhibitors in response to the reduced starch as a result of drought stress [36]. The growing location had a significant impact on the amounts of CM3 WTAI [27,32]. All these studies confirmed what was observed in the present study that, besides genetic factors, also the growing environment and the climatic conditions influence the ATI activity in the wheat grains.

The inhibitory activities were measured in this study against mammalian enzymes. It is known from the literature that each type of ATI has a different specificity and that it can be differentially activated by different types of stress. Further studies are needed to understand possible mechanisms able to modulate the enzymatic activity in response to different types of biotic stress.

### 2.5. ATI-Inhibitory Activity and Human Nutrition

ATIs have also a relevant place in human nutrition. Besides their role in allergies and in the etiology of celiac disease and non-celiac wheat sensitivity [6], ATIs can act indirectly by suppressing the activity of digestive enzymes, namely trypsin and alpha-amylase, with two independent binding sites [4,37] and with consequent impact on human health.

Inhibition of alpha-amylase activity leads to accumulation of undigested and unabsorbed starchy constituents which are fermented in the distal gut by the microbial flora and can cause mild to moderate symptoms, including flatulence and bloating, in sensitive individuals to carbohydrate malabsorption [38]. These symptoms are also reported by non-celiac wheat sensitivity patients after consuming wheat products [39]. However, the alpha-amylase inhibitory activity of wheat-based food has significant relevance for its dietary and therapeutic support to potentially maintain postprandial glucose homeostasis, which is essential for prevention and overall management of early-stages type 2 diabetes [40].

High trypsin inhibitory activities can be negative from a nutritional point of view because these can limit the absorption of protein during food assumption which decreases its nutritional usefulness [41]. It was previously described that an excessive amount of trypsin inhibitors in food can progressively lead to pancreas hyperplasia or hypertrophy and is correlated to a higher risk of pancreatic cancer. Imbalanced and uncontrolled proteolysis can also lead to tissue damage such as inflammation, hypertension, gastric ulcer, tumor growth and metastasis [42]. For these reasons, protease inhibitors are considered “antinutritional compounds”, due to their inhibitory activity on digestive enzymes in humans and animals.

According to the results of this study, the genotypes which showed in general the lowest inhibitory activities against both alpha-amylase and trypsin and could be better tolerated by people suffering from wheat sensitivities were turanicum and Alzada, while the highest activities were shown by the hexaploid Judee, Turkey Red and Marquis. In line with these results, consumption of T. turgidum ssp. turanicum wheat-based products has been previously shown to improve symptoms in people suffering from irritable bowel syndrome [43]. Moreover, considering the potential effect on glycemic control, emmer cultivated in Italy showed the best combination for diabetic people with high alpha-amylase inhibitory activity, but low trypsin inhibitory activity. The evidenced differences, even if they require confirmation in further studies also in vivo, can be useful information for the selection of the better genotypes for specific food needs.

The effect of ATIs on the human organism highly depends on how these proteins are processed by the digestive enzymes. ATIs are members of the prolamin superfamily, and these proteins are characterized by having high resistance to digestion and denaturation [7]. However, a recent study showed that the ATIs found in two varieties of einkorn were completely digested by gastrointestinal enzymes with the result that these einkorn samples failed to induce innate immune response in vivo, unlike the modern common wheat varieties included in the same study [44]. So, the effect of ATIs in the human body highly depends on the specific variety of wheat selected as a source of these proteins. These findings suggest that the gut digestion could neutralize the high trypsin inhibitory activity of einkorn shown in this study and, consequently, its potential negative effect on the human body. It could be hypothesized that the result of an in vivo effect of digestive enzymes could evidence differences among wheat genotypes not highlighted in vitro in the present study. Therefore, additional in vivo studies are needed to deepen knowledge on the differential effect of gastrointestinal enzymes on ATIs from different wheat genotypes and the resulting impact on human health.

Recent studies showed that activity and pro-inflammatory properties of ATIs can be lost also as a consequence of food processing, such as bread making, sourdough fermentation and baking [45,46] as well as pasta cooking [47]. Moreover, little residual inhibitory activity towards alpha-amylase from human saliva was detected in commercial and laboratory-made cereal foods [48]. So, both the alpha-amylase and trypsin inhibitory activities should be tested for the resistance to the digestive enzymes and to food processing to assess their residual activity before drawing final conclusions about the impact of a specific wheat genotype on human health.

## 3. Materials and Methods

### 3.1. Grain Samples

The present study included ten selected accessions belonging to different *Triticum* species, i.e., *T. monococcum* L. subsp. *monococcum* (2n = 2x = 14 chromosomes, AA genome), *T. turgidum* L. subsp. *dicoccum*, *T. turgidum* L. subsp. *turanicum*, *T. turgidum* L. subsp. *durum* (all 2n = 4x = 28 chromosomes, AABB genomes), *T. aestivum* L. subsp. *aestivum*, *T. aestivum* L. subsp. *spelta* (all 2n = 6x = 42 chromosomes, AABBDD genomes). The selection of these genotypes was made to include different wheat species with a different genome composition (diploid, tetraploid, hexaploid) and with a different year of release, when available. A full description of these wheat accessions was reported in Simonetti et al. (2022) and in Table 5.

The ten selected wheat samples were grown under organic farming management during three consecutive crop years at two locations: at the Quinn Farm & Ranch located near Big Sandy, Montana, USA (48°02′19″ N, 110°00′55″ W, 921 m a.s.l.) and at the experimental farm Podere Santa Croce located at Argelato, Bologna, Italy (44°39′57″ N, 11°19′43″ E, 16 m a.s.l.). The field trial soil in the USA was sandy–loam while in Italy was clay–loam. The soil parameters prior to sowing are shown in Table 6.

The three crop years were 2015–2016; 2016–2017; 2017–2018 in the USA and 2016–2017; 2017–2018; 2018–2019 in Italy. In the USA, the wheat samples einkorn, emmer, turanicum, Peliss, Alzada, Marquis and Vida were cultivated on a spring cycle (planted in May and harvested in August), while spelt, Turkey Red and Judee were cultivated on a winter cycle (planted in September and harvested in early August). In Italy, all ten wheat samples were planted in November and harvested in July. The meteorological data (temperature and rain) were recorded at both sites from the National Center of Environmental Information [49] for the American location and from Arpae Emilia Romagna [50] for the Italian location.

Grain samples from different genotypes were tested for their protein contents by employing the Foss Infratec 1229 NIT spectrophotometer (Global calibration No. WH000003).

The grain samples were milled with the domestic stone mill (100% flour extraction) (Billy 200, Hawos Kornmülen gmbh, Germany), and the flour obtained was used for the assessment of the mycotoxins content and for the enzymatic tests.

### 3.2. Assessment of Mycotoxin Levels

The content of deoxynivalenol (DON) was determined with an enzyme-linked immunosorbent assay using the AgraQuant^®^ Deoxynivalenol ELISA test kit (Romer Labs GmbH, Tulln, Austria) and following the manufacturer’s instructions. The color produced was read at 450 nm with the Labsystems Multiskan MS Microplate Reader (Labsystems, Ottawa, ON, Canada).

### 3.3. Enzymatic Assays

The whole wheat flour samples previously obtained from the ten wheat genotypes cultivated for three consecutive years in two growing areas were extracted in duplicate and, two replicates from each sample extract were tested independently in the following enzymatic assays.

#### 3.3.1. Alpha-Amylase Inhibitory Activity Assay

In duplicate, the flour samples were extracted using cold water. Briefly, 100 mg of each of the flour samples was homogenized with 500 μL of distilled water using an automatic shaker at 160 rpm for 30 min. The samples were then centrifuged at 7000 rpm for 5 min, and 400 μL of supernatant was collected. The remaining pellet was again homogenized with 400 μL of distilled water at 160 rpm for 5 min, and after a centrifugation at 7000 rpm for 5 min, 350 μL of supernatant was collected and added to the first supernatant. Then, the supernatant was heated at 70 °C for 20 min to inactivate endogenous enzymes, diluted 1:100 in distilled water and used the same day for alpha-amylase inhibitory activity assessment.

In duplicate, 1.7 μL of each sample extract was transferred to a 96 well flat-bottom clear plate and incubated for 10 min at 25 °C with 48.3 μL of amylase assay buffer (Sigma–Aldrich Co., Oakville, ON, Canada) containing 20 mU of alpha-amylase from human saliva (Sigma–Aldrich Canada Co.). The control was the amylase assay buffer containing 20 mU of alpha-amylase from human saliva without the addition of the sample extract. The amylase activity was tested with the Amylase Activity Assay Kit (Sigma–Aldrich Canada Co.) following the manufacturer’s instructions. This test is based on the production of a colorimetric product at 405 nm (*p*-nitrophenol) which is proportional to the amount of the substrate (ethilidene-pNP-G7) hydrolyzed by the amylase. The absorbance was measured at 405 nm with the Labsystems Multiskan MS Microplate Reader. The inhibitory activity of the extracts was calculated as a percentage with respect to the control according to the following formula:

inhibition % = [(B_control_ − B_sample_)/B_control_ × 100] (1)

where B is the amount (nmol) of colorimetric product generated between T_initial_ and T_final_ and calculated using the standards provided by the kit; T_initial_ is the first absorbance measure taken 3 min after the addition of the substrate; T_final_ is the absorbance measured when the most active sample was close to but not exceeding the end of the linear range of the standard curve.

#### 3.3.2. Trypsin Inhibitory Activity Assay

In duplicate, the flour samples were extracted using a sodium acetate buffer solution with a pH of 3.8 and ionic strength of 0.02 N as reported by Chang and Tsen [28]. Morever, 1 g of each flour sample was homogenized with 15 mL of solvent using an automatic shaker at 160 rpm for 1 h. The samples were then centrifuged at 10,000 rpm for 30 min at 4 °C, and 800 μL of supernatant was collected and used the same day for trypsin inhibitory activity determination.

In duplicate, 2 μL of each extract was transferred to a 96 well flat-bottom clear plate and incubated for 20 min at 25 °C with 48 μL of trypsin assay buffer (Sigma–Aldrich Canada Co.) containing 8 U of trypsin from bovine pancreas (Sigma–Aldrich Canada Co.). The control was the trypsin assay buffer containing 8 U of trypsin from bovine pancreas without the addition of the sample extract. The trypsin activity was tested with the Trypsin Activity Colorimetric Assay Kit (Sigma–Aldrich Canada Co.) following the manufacturer’s instructions. This test is based on the production of a colorimetric product at 405 nm (*p*-nitroaniline) which is proportional to the amount of the substrate hydrolyzed by the trypsin. The absorbance was measured at 405 nm as described for the alpha-amylase inhibitory activity assay (see Section 3.3.1).

#### 3.3.3. Statistical Analysis

The general linear model (GLM) was used to assess the variance significance for the fixed (genotype) and the random (year) factors, as well as their interactions, for all measured variables. Tukey’s (HSD) test was used to determine differences between means at *p* < 0.05. Analysis was performed using IBM SPSS Statistics 25 software. The analysis of correlation was performed using STATISTICA Software v. 7.1, (StatSoft, Tulsa, OK, USA).

## 4. Conclusions

The tested in vitro inhibitory activities showed high variability among the ten wheat genotypes analyzed, and the contributions of the genotype and the crop year were significant for both the alpha-amylase and the trypsin inhibitory activities in the two growing areas. In general, the hexaploid wheat genotypes showed the highest ATI inhibitory activities, and it was not possible to highlight any trend in ATI activities as function of release year of wheat samples. Einkorn had a peculiar behavior showing the lowest alpha-amylase inhibitory activity, but the highest trypsin inhibitory activity. The tetraploid turanicum and Alzada wheat samples showed an interesting profile, with the lowest inhibitory activities against both alpha-amylase and trypsin The two inhibitory activities were differently affected by the growing conditions and were negatively correlated with the protein content.

Although the number of samples tested was limited, the information collected may be important to understand the extent of the variation of ATI inhibitory properties in relation to the wheat genotype and the growing environment and its impact, if any, on human health and nutrition.

## Figures and Tables

**Table 1:** Protein and DON mean content of the wheat samples grown in the USA and in Italy. Standard deviation (SD) is reported in brackets in the table.

USA	2015–2016		2016–2017		2017–2018	
Wheat Genotype	Protein (g/100g dm)	DON (ppb)	Protein (g/100g dm)	DON (ppb)	Protein (g/100g dm)	DON (ppb)
Einkorn	26.7 (1.0)	<200	20.8 (1.1)	<200	20.8 (1.1)	<200
Emmer	Sample not available		19.7 (0.6)	<200	19.1 (0.8)	<200
Turanicum	19.4 (0.6)	253 (18.4)	15.8 (0.6)	<200	17.1 (0.4)	<200
Peliss	Sample not available		Sample not available		18.5 (0.7)	<200
Alzada	24.7 (1.0)	376 (22.6)	17.3 (1.1)	<200	19.7 (0.7)	<200
Spelt	14.8 (0.4)	204 (5.7)	18.6 (0.1)	<200	19.5 (0.7)	<200
Turkey Red	14.6 (0.4)	<200	17.0 (0.7)	<200	16.8 (0.1)	<200
Judee	13.7 (0.1)	<200	17.0 (0.3)	<200	16.4 (0.6)	<200
Marquis	22.3 (1.4)	<200	18.5 (0.6)	<200	19.2 (0.3)	<200
Vida	18.6 (0.8)	296 (8.5)	17.8 (0.3)	<200	17.9 (0.3)	<200
**Italy**	**2016–2017**		**2017–2018**		**2018–2019**	
**Wheat Genotype**	**Protein** **(g/100g dm)**	**DON** **(ppb)**	**Protein** **(g/100g dm)**	**DON** **(ppb)**	**Protein** **(g/100g dm)**	**DON** **(ppb)**
Einkorn	15.2 (0.3)	<200	14.0 (0.7)	<200	17.3 (0.4)	<200
Emmer	12.3 (0.4)	<200	12.5 (0.7)	<200	11.1 (0.1)	219 (12.7)
Turanicum	12.7 (0.3)	<200	12.8 (0.3)	593 (18.4)	10.3 (0.4)	969 (12.7)
Peliss	12.3 (0.4)	<200	13.2 (0.6)	383 (18.4)	10.1 (0.1)	342 (17.0)
Alzada	12.6 (0.1)	299 (19.1)	14.2 (0.4)	547 (23.3)	11.5 (0.7)	1122 (45.3)
Spelt	12.0 (0.1)	<200	13.8 (0.3)	<200	14.0 (0.7)	260 (14.1)
Turkey Red	11.3 (0.7)	<200	11.4 (0.1)	<200	10.1 (0.1)	271 (15.6)
Judee	11.4 (0.4)	<200	11.3 (0.1)	<200	10.1 (0.1)	920 (14.1)
Marquis	12.5 (0.1)	<200	12.9 (0.6)	<200	12.5 (0.6)	<200
Vida	12.7 (0.3)	<200	13.3 (0.4)	<200	13.1 (0.1)	373 (11.3)

**Table 2:** Mean values (%) of the alpha-amylase inhibitory activity for wheat genotypes, years and growing areas. Different letters indicate statistically significant different means for *p* < 0.05. ** *p* < 0.01.

USA		Italy	
Year (Y)	**	Year (Y)	**
2015–2016	40.83 (b)	2016–2017	53.43 (a)
2016–2017	56.26 (a)	2017–2018	47.06 (b)
2017–2018	49.75 (a)	2018–2019	38.52 (c)
**Genotype (G)**	**	**Genotype (G)**	**
Einkorn	13.76 (d)	Einkorn	1.12 (f)
Emmer	56.62 (ab)	Emmer	65.42 (a)
Turanicum	39.6 (c)	Turanicum	44.14 (cd)
Peliss	59.73 (ab)	Peliss	48.80 (c)
Alzada	47.61 (bc)	Alzada	33.28 (e)
Spelt	63.15 (a)	Spelt	59.95 (b)
Turkey Red	66.63 (a)	Turkey Red	56.42 (b)
Judee	60.50 (a)	Judee	57.47 (b)
Marquis	49.24 (bc)	Marquis	55.00 (b)
Vida	45.35 (bc)	Vida	41.77 (d)
**G × Y**	**	**G × Y**	**

**Table 3:** Mean values (%) of the trypsin inhibitory activity for wheat genotypes, years and growing areas. Different letters indicate statistically significant different means for *p* < 0.05, ** *p* < 0.01. ns, not significant.

USA		Italy	
Year (Y)	**	Year (Y)	**
2015–2016	63.31 (a)	2016–2017	42.53 (a)
2016–2017	43.72 (c)	2017–2018	39.57 (b)
2017–2018	49.04 (b)	2018–2019	35.47 (c)
**Genotype (G)**	**	**Genotype (G)**	**
Einkorn	100.00 (a)	Einkorn	100.00 (a)
Emmer	60.55 (d)	Emmer	19.34 (e)
Turanicum	23.08 (h)	Turanicum	20.93 (d)
Peliss	18.61 (i)	Peliss	18.77 (e)
Alzada	30.45 (f)	Alzada	23.30 (d)
Spelt	39.46 (e)	Spelt	36.18 (c)
Turkey Red	66.54 (c)	Turkey Red	49.27 (b)
Judee	71.06 (b)	Judee	51.70 (b)
Marquis	59.00 (d)	Marquis	49.76 (b)
Vida	27.26 (g)	Vida	22.64 (d)
**G × Y**	**ns**	**G × Y**	**ns**

**Table 4:** Analysis of variance: percentage of mean square obtained from the ANOVA highlighting the contribution of genotype and crop year and their interaction to the variability of alpha-amylase (a) and trypsin (b) inhibitory activity. * *p* < 0.05, ** *p* < 0.01. ns, not significant.

(a)			
	Year (Y)	Genotype (G)	G × Y
**USA**	37.5 **	55.3 **	7.2 **
**Italy**	34.1 **	62.9 **	2.3 **
**(b)**			
	**Year (Y)**	**Genotype (G)**	**G × Y**
**USA**	32.8 **	64.7 **	1.8 ns
**Italy**	6.0 *	91.5 **	1.4 ns

**Table 5:** List of wheat genotypes used in this study.

Ploidy Level	*Triticum* Species	Genotype	Year of Release	Classification	Name Used in This Study
Diploid	*T. monococcum* L. subsp. *monococcum*	Local landrace ^a^	/	Hulled, ancient wheat	Einkorn
Tetraploid	*T. turgidum* L. subsp. *dicoccum*	Local landrace ^a^	/	Hulled, ancient wheat	Emmer
	*T. turgidum* L. subsp. *turanicum* *	QK-77 ^b^	/	Naked, ancient wheat	Turanicum
	*T. turgidum* L. subsp. *durum*	Peliss ^c^	1900	Naked, heritage durum wheat	Peliss
	*T. turgidum* L. subsp. *durum*	Alzada ^a^	2004	Naked, modern durum wheat	Alzada
Hexaploid	*T. aestivum* L. subsp. *spelta*	Local landrace ^a^	/	Hulled, ancient wheat	Spelt
	*T. aestivum* L. subsp. *aestivum*	Turkey Red ^d^	1873	Naked, heritage hard red common wheat	Turkey Red
	*T. aestivum* L. subsp. *aestivum*	Judee ^a^	2011	Naked, modern hard red common wheat	Judee
	*T. aestivum* L. subsp. *aestivum*	Marquis ^a^	1913	Naked, heritage hard red common wheat	Marquis
	*T. aestivum* L. subsp. *aestivum*	Vida ^a^	2006	Naked, modern hard red common wheat	Vida

* sold under the KAMUT^®^ brand. KAMUT^®^ is a registered trademark of Kamut International, Ltd., and Kamut Enterprises of Europe, bv. Provenance: Montana State University, Montana, USA (a); Kamut International, Montana, USA (b); University of Saskatchewan, Canada (c); Hartland Mills, Kansas, USA (d).

**Table 6:** Soil parameters prior to sowing for years and growing areas (a), Italy; (b) USA. The soil parameters were supplied by Agriparadigma Laboratories (Ravenna, Italy).

(a)			
Soil Characteristics before Sowing	2016–2017	2017–2018	2018–2019
pH	7.6	6.6	7.6
Organic matter (% dm)	1.2	3.3	3.7
Total N (‰ dm)	0.7	1.4	1.5
C/N ratio	9.7	13.6	14.7
Assimilable *p* (mg/kg dm)	16.9	8.3	13.8
Assimilable K (mg/kg dm)	190.1	178.5	182.3
**(b)**			
**Soil Characteristics before Sowing**	**2016–2017**	**2017–2018**	**2018–2019**
pH	7.3	7.0	7.4
Organic matter (% dm)	1.5	1.8	2.4
Total N (‰ dm)	0.9	0.8	0.5
C/N ratio	9.6	13.1	27.9
Assimilable *p* (mg/kg dm)	14.2	12.1	11.6
Assimilable K (mg/kg dm)	261.1	239.4	211.9

## Data Availability

Not applicable.
